# Medical Report of the Fever Hospital, Dublin

**Published:** 1830-10-01

**Authors:** 


### MEDICAL REPORT OF THE FEVER HOSPITAL, DUBLIN. By Dr. *J. O'Brien*.

IN the annual report of the above hospital for 1826, the author had occasion to describe the formidable epidemic of that period—an epidemic the most extensive as to numbers, which had ever previously visited that city. It was computed that 50,000 persons, that is, about a sixth part of the population, had been involved in the calamity on that occasion, of whom, from three to four thousand died.

"Since that period a revolution not unusual in Epidemic maladies, and similar to those which mark the vicissitudes of other great natural phenomena in which extremes often follow each other in rapid succession, has occurred with respect to Fever in this metropolis. Not only has this disease fallen below its ordinary numerical standard, but at particular moments, it appeared to be altogether evanescent and extinct. On several occasions the number of cases of Typhoid Fever in this hospital, did not exceed half a dozen, and I have reason to believe, that a few occasions occurred, on which not a single case of Typhoid Fever was to be found in our wards."

In respect to the etiology of fever, our author completely overturns the medico-political doctrines of Dr. Corrigan, which have been bruited about in the Lancet and the newspapers—namely, that *want* is the cause of the Irish epidemics.

"There is one fact, however, which he thinks it important to notice, and which, he conceives to be a decisive refutation of that opinion, which would attribute the generation of Epidemic Fever to the sole and exclusive agency of public distress and famine;—namely, that during the period of Epidemic intermission, if we may be allowed the phrase, just now described, when this disease had nearly disappeared from amongst us, public distress and suffering had never previously, perhaps, attained to so great a height in Dublin. This unhappy state of things is so well authenticated by repeated public meetings, and repeated claims on public charity, that it would be quite superfluous to offer further proofs of its existence.

It is well known that the great mass of the humbler population in the district immediately contiguous to this hospital, and from which its wards are principally supplied, have, after suffering the most afflicting distress, been fed for months back from the scanty gleanings of public charity. The Author, however, is far from denying the powerful agency of want and misery in generating Epidemic

Fevers; he has ever regarded those evils, in conjunction with certain moral habits, which he looks upon as their natural and inevitable consequences, to be the chief, *the great*, he would say it emphatically, *predisposing causes* of Fever in this country; but he holds the opinion that these evils alone are incapable of generating Continued Fever in any individual instance, much less in its epidemic form, and that to produce this effect, the combined agency of another set of causes, which, in medical language, we call exciting causes, is indispensably necessary. This latter class of causes will operate with tenfold effect upon an impoverished and enfeebled multitude, when present, but when they are absent, we are instructed by the events of the past year, distinguished at once for an extraordinary immunity from Fever, and extraordinary public distress, that want and misery are incapable of themselves, of producing Epidemic Fever." 7.

Dr. O'Brien next introduces a series of cases from the journals of the hospital, amounting to sixteen, exhibiting, as he expresses it, "a *lively* portrait of the different types of fever which prevail in Dublin." The first four patients present examples of primary gastro-enteritis, "or that disease which has proved a false light to M. Broussais." The fifth case exhibits an example of complication of gastro-enteritis with typhoid fever—and the remaining cases afford illustrations of various types of typhoid fever, "from the mildest to the most intense and malignant form of the disease." We shall give the following general conclusions in the author's own words, as they cannot be abbreviated without injury.

"First.—That there exists a primary Gastro-enterite, attended by a Fever of a peculiar kind, approximating in some respects to the Typhoid character, like all intense phlegmasiæ of the Gastro-intestinal canal, yet differing from Typhus by some obvious and striking properties.—The following is the train of symptoms peculiar to this disease, viz.—Pain, uneasiness, and generally fulness of the epigastrium, or abdomen, or both aggravated by pressure, and accompanied by headache, nausea, or retching, and, in many instances, by frequent vomiting, particularly after the introduction, even of the smallest quantity of fluid or solid aliment, into the stomach. The appearance of the tongue is peculiar and characteristic; it is either of a vivid or dark red colour, over its entire surface, or it is red at the edges and point, but covered with a dark white fur in the centre, through which specks of red are occasionally visible; the centre, however, is also frequently brown, or even of a yellowish hue, whilst the edges are dark red, as above described, and the papillæ all over the surface unusually prominent; and this organ, on the whole, presents a more striking appearance of irritation and sub-inflammation in this disease than in any other type of Fever.—The pulse is usually deficient in fulness; it is small, frequent, and compressible, and approximates more to the Typhoid than the Synochoid character. It is also accompanied by a lower temperature of the skin; and, in a word, displays none of the signs of that strong re-action, which marks the early stage of Synochus. It is distinguished, however, from Typhus by the comparative mildness of the cerebral affection; the author has, indeed, been frequently surprised at the clearness and integrity of the intellectual faculties, in the midst of that extreme depression of the muscular powers which characterises this type of Fever. This disease is slow and gradual in its access as well as its progress; the patient feels himself ill for some time, affected with loss of appetite, costive bowels, uneasiness, and occasional twitches of pain at the epigastrium and in the abdomen, which continue until the febrile movement is developed, when the train of symptoms before described, sets in with all its violence. The progress is also remarkably slow, the disease being frequently protracted to the sixth or seventh week before convalescence takes place. It is further distinguished from Typhus by the absence of petechiæ, a black crust on the tongue, or black sordes of the teeth and gums, which the author has never observed in any of the clearly marked cases of this disease he has witnessed. The bowels are either constipated, or too relaxed, and occasionally these two states alternately succeed each other.—The abdomen is tumid, resisting and tender to the touch, when pressure is employed externally;—the sleep is uneasy, interrupted, and delirious; but when awake, the patient seems to suffer little diminution of his intellectual powers.

As a further proof of the real nature of this affection, it may be stated, that the author has invariably observed that, in proportion as the abdominal symptoms were mitigated or subdued, the affection of the head and the febrile symptoms suffered a simultaneous mitigation or removal. The colour of the skin in this disease is commonly one of the shades of yellow; occasionally the tint is deep and dark, as in the case of Kitts (No. 1), where it approached to one of the lighter shades of mahogany.—The intense bright yellow colour of the skin, peculiar to jaundice, and, we presume, to yellow Fever, has not occurred in this Hospital since the Epidemic Fever of 1826; but, from the author's recollection of the cases which then occurred, he is inclined to consider them as modifications of the disease we have been considering.

"Secondly.—The disease now described may be secondary, that is, may supervene on Typhoid Fever, a predisposition being probably formed by previous disorder of the stomach and alimentary canal, functional or organic, or by the prevalence of that epidemic constitution or Malaria which disposes to diseases of the stomach and bowels, as Cholera, Dysentery, &c. This adjunct to Typhoid Fever may occur at an early period of the disease, but it is more frequently observed to accompany the advanced stages.

In those cases, we are taught by numerous dissections made by modern pathologists, that the principal, in many instances, the sole seat of disease, is the lower part of the *ileum*, near its junction with the *cæcum*, which is probably to be attributed to the densely glandular structure of this part of the intestine, and partly, as we believe, also to its inferior situation, which favours the accumulation of acrid secretions in this part. In a disease like Typhus, where the sensibility is greatly impaired, or even destroyed altogether, this affection may exist, without being felt or complained of by the patient; but it will very seldom, indeed, fail to be detected by careful examination externally, or by diarrhœa, or a tympanitic state of the abdomen—which latter are its appropriate signs, when the patient is in a state of *coma* or insensibility. Another of its symptoms, more rare than those above-mentioned, is hæmorrhage from the bowels, which, if superadded to a tympanitic state, presents the most intense and hopeless form of this affection. With respect to the colour of the alvine discharges in this affection, it is stated by Dr. Bright that they are generally ochre-coloured; but the author has more frequently seen them of a dark or mud colour—yet he has also frequently observed them as described by Dr. Bright.

Thirdly.—The author is of opinion that there are good grounds in nature for dividing Idiopathic continued Fever into two great classes, which become the foundation of important practical indications, viz.: Inflammatory Fevers (Synochæ), and Typhoid Fevers (Synochus and Typhus); between the first and second of which, as formerly observed of the second and third, various intermediate shades of type occur, the allocation of which to this or that genus or class, it will be difficult to determine. Again, he infers that in the first class (Synocha), the Morbific cause of Fever exercises its agency principally over the heart and arterial system, whilst the sensorium and nervous system enjoy a comparative exemption from its influence. Further, that the operation of the Morbific cause in Typhoid Fever is directed primarily and essentially against the brain and nervous system (including the spinal chord), and through that system against the heart and arteries, and their capillary extremities. That the various modifications of class and species, arising out of the two great divisions above-mentioned, may depend on specific modifications in the Morbific cause itself, or in the original conformation or constitution of the individual who is the recipient of the Morbific impression; but that the relation between cause and effect here is as yet far removed from our comprehension. We know, however, that in every case of Typhoid Fever the prominent features of the disease, from first to last, and the character of its symptoms, are nervous, modified by the various degrees of arterial and vascular action by which they are accompanied." 54.

If we examine the first of the two species of typhoid fever (synochus) we shall observe the phenomena to succeed each other in the following order.

"First, a stage of nervous and vascular depression; secondly, a stage of vascular excitement or reaction; and thirdly, a stage of universal exhaustion and debility, announcing a more complete depression of the nervous, vascular and muscular powers than in the first stage. In the perfect Typhus again, the whole series of phenomena exhibit only increasing degrees of nervous, vascular and muscular depression; the power of arterial reaction is annihilated, and the state of the system approaches to that of general paralysis." 55.

This leads our author to a consideration of the physiological condition of the brain itself in this disease. In every case, he thinks, there is a determination of blood to the head, sufficiently manifested by the red and injected eye, the burning forehead, &c. Whether the state of the brain be inflammation or congestion, it is almost needless to inquire, because we do not precisely know what the pathological character of either of these states is.

"We know, however, with certainty, and to this fact, perhaps, our knowledge in every case of inflammation is limited, that the ordinary condition of the brain in Typhoid Fever is that of vascular fulness and distention; but beyond the expression of this simple fact, strict philosophy will not permit us to proceed. Dissection may, indeed, occasionally discover the vestiges of acute inflammation in the brain; but we believe, in the majority of cases, it has failed to detect it. This condition of the brain, the author holds to be consecutive, not primary, in the morbid series, which constitutes the disease, but when once fully established, it becomes itself a new source of morbid actions, re-acting on the sensorial disorder which produced it; and thus, by its direct and reflex influence, producing the characteristic phenomena of the disease. But this condition, which may be called Typhoid Inflammation, may also be propagated to other organs essential to life, as the lungs, stomach, &c.; and we are instructed by dissection, that nearly the whole of the mucous surface, or internal lining of the body, is in a state of vascular distention in Typhoid Fever." 56.

We are not, as yet, sufficiently advanced in the science of the animal fluids to determine what part the blood may play in the generation of fever; but so far as experiments have gone, it has been found but little altered in the first days of the disease.

"We know, however, with certainty, that in a short, but indefinite period after the formation of the disease, the blood and other animal fluids suffer a manifest alteration in their physical properties; and thus, in their turn, become a part of the morbid circle which constitutes the disease." 57.

This is a rational supposition. Our author conceives that the followers of Broussais err chiefly by the indefinite application and the universality of their doctrine, extending it to all classes and species of fever. The following summary of the treatment of fever, founded on seven years' experience in a public institution, we shall give in the author's own words.

SUMMARY.

"The great object of establishing correct principles is to guide us to a safe and successful practice, to rescue the victim from the grasp of death, and restore him to health; this is the end of all our reasonings, our labours, our anxious solicitude. But, alas! have we arrived at that point of certainty in our principles, that would enable us to rely on them with confidence as fixed rules of practice?—Can he who contemplates the divisions which embarrass the science of Pyretology, the numerous theories, and conflicting opinions, which distract its cultivators, lay his hand to his breast and say, that any theory of Fever, which has hitherto been proposed, furnishes a principle, which he would at once apply without fear and hesitation to the treatment of fever? However partial he may feel to the views, a miniature of which, he has endeavoured to lay before the reader, the author is compelled by truth and candour to declare, that he would answer in the negative.

There is no part of the philosophy of Fever, if I may use the phrase, which has been treated with more levity, and disregard of the strict rules of induction than its treatment; and yet this is the most important of all, the great problem to whose solution all our researches are directed. This is the grand error of speculative and theoretic reasoners; they first form their principles, and then deduce the practice, and too often apply it without hesitation or remorse; whereas, strict philosophy requires that they should first establish the practice, and afterwards deduce the principles.—In fine, our proposition is, that every man should form his practice, not from preconceived opinions, not from delusive theories, but from a multitude of facts and experience.

Let the advocate of primary inflammation, who commands us to abstract sixty ozs. of blood in the first stage of Typhoid Fever, first bring forward his individual cases, numerous and authentic, in confirmation of the success of this practice, and then if we are satisfied with their number and authenticity; if, in fine, they stand the test of rigorous examination, we shall admit that he is warranted in his conclusions to the extent of his experience. But we should afterwards take care to make a minute comparison of that experience with the experience of others, and with our own. The patrons of Humoralism on the other hand, we would address in a similar strain, and entreat them to bear in mind, the uncertainty, to say the least of it, of this doctrine, and to recollect, that where human life is the subject of experiment, and must be the sacrifice of error, ambiguous principles should never become the basis of practice.

The author shall now proceed to state the result of seventeen years' experience in the treatment of Fever, in that Hospital whose report he now writes.

The primary Gastro-enterite is rarely a fatal disease, I mean in its ordinary form of prevalence in Dublin. In its mildest varieties are included all those cases of mild Fever which are commonly attributed to indigestion and disordered bowels, and which are the peculiar plague of infancy and childhood.

These complaints are, for the most part, easily removed by removing the irritation which produces them, that is, by unloading the alimentary canal of its acrid contents, which is best effected by a few gentle purgatives, among which a small dose of calomel or some other mercurial will generally be necessary. In the severer forms of the disease in which M. Broussais, we think, with justice includes 'delirium tremens,' the most efficacious of all remedies, according to the author's experience, is local or capillary blood-letting. Occasionally, when the accompanying Fever assumes the Synochoid character, the author commences his treatment by taking twelve or fourteen ounces of blood from the arm; a measure which will also be generally advisable, when the pain of the epigastrium and abdomen are very acute, and increased by slight external pressure. The remainder of the treatment may be conducted by the repeated application of leeches to the epigastrium, or abdomen, according to the nature of the case, until all pain and tension be removed. As the number of leeches at each application may be considered a point of importance, it may be stated that the maximum number in the four first cases recorded above, and which were cases of great severity, was twelve at each application; and even from this number (which some will probably consider too small,) the author has invariably found a decided benefit. After the leeches, it is usual to apply warm stupes or an emollient poultice over the part most affected. Next in efficacy to local blood-letting, we would place laxatives;—irritating purgatives are not applicable here, though we by no means sympathise in the horror which the disciples of M. Broussais entertain against this class of medicines, reasoning, not from experience, but from the prejudices of theory;—indeed we entertain little doubt, that as the disease is frequently produced by the omission, so it is protracted by the neglect, or fear of purgatives.

Rhubarb and manna were the favourite purgatives of Rœderer and Wagler, and accordingly we believe the infusion of rhubarb and manna to be, by its mildness and efficacy, well suited to this disease. In hospital practice we use small doses of castor oil and tincture of rhubarb, or more frequently rhubarb alone, suspended in some aromatic vehicle. A pill, composed of rhubarb, Dover's powders, and a moderate quantity of the *hydrargyrus cum creta*, or of blue pill, is a combination which the author frequently employs in this disease, and which he has found to fulfil the indications required in this complaint of a soothing and mild laxative.

The next class of medicines applicable to this disease is mercurials, and of these the particular form which we prefer is calomel and opium. This medicine has been found often successful in allaying irritation of the stomach and vomiting, when means above recommended had failed in producing this effect. The author has not tried mercurial friction, but he has no doubt that particular cases may occur in which this may be the most advisable mode of administering the remedy. Whatever form of mercury is used, it will be necessary to carry it to the extent of a slight salivation, before the full effect is produced. One of the most distressing symptoms in this complaint is nausea and vomiting, which incessantly harass the patient. When the measures above pointed out have failed to remove this symptom, we have applied a mustard poultice or a blister to the epigastrium, which have seldom failed to remove it. In those cases Dr. Bright recommends the *carb. magnesiæ* internally, and small draughts of soda water, with a tea-spoonful of brandy. When the inflammatory symptoms have removed or mitigated, we consider opium as a medicine of considerable value in this complaint. In the case of an esteemed medical friend, (himself an experienced Physician,) whom the author attended, while labouring under a very protracted form of this disease, it was thought advisable to administer opium to allay abdominal pain and irritation, and the Acetate of Morphia was selected as least likely to disagree with the stomach;—so decided was its effect, and so prompt and permanent the relief, that the gentleman assured my colleagues in attendance and myself, 'that we had hit upon the true remedy at last.' When however, Diarrhœa becomes a prominent symptom of the disease, opium, or any other medicine calculated to constipate, should be administered with caution, as we consider moderate diarrhœa a sanative process in the inflammations of the gastro-intestinal mucous membrane. In such cases our object is to moderate, not suppress this discharge; and for this purpose we have found Dover's powders and rhubarb one of the best remedies, in conjunction with gummy and mucilaginous diluents. Secondary inflammation of the mucous or serous structures of the lungs are by no means an unfrequent complication in this disease; of which examples occur in the cases of Somers and Duffy, above described. Such cases are to be treated according to the ordinary method by general blood-letting, if the strength of the patient permit; but if not, by the local abstraction of blood, blisters, and expectorants, as ipecacuanha, squill, &c. Such is the mode of treatment which had been adopted in the cases above described, and they recovered under very unpromising circumstances.

The secondary Gastro-enterite which supervenes on Typhoid Fever, is to be treated in the manner now described with respect to the primary disease, with the exception of general blood-letting, which is never admissible; the disease must be encountered, in this case, by the repeated application of leeches, laxatives, and mucilaginous diluents, and the other remedies above described. Of hæmorrhage from the bowels, we have one example in the cases above detailed, and it is by no means an infrequent occurrence in this form of the disease; it is even common for slight hæmorrhage to continue after the fever has disappeared, and to protract the convalescence beyond its natural period. In all the cases of this description which occurred in the author's ward, the means above described were attended with complete success." 63.

The author then approaches the last and most difficult subject of investigation—the general treatment of typhus fever. This he first considers abstractedly from its complication with various inflammatory affections—secondly, in relation to those complications themselves. Dr. O'Brien adverts to the three stages of typhoid fever—nervous depression—arterial excitement—final exhaustion. The following critique on Dr. Smith is deserving of a place here, as it bears on some observations made by ourselves in the review of Dr. Smith's work.

"Suppose a patient presented to us in the first stage, that is, in the first two or three days of Fever, with a pale shrunk countenance, a shrivelled skin, a general feeling of coldness, a heavy and dejected air, a faltering voice, head-ache, anorexia or nausea, or vomiting—what is to be done? Bleed, says Dr. Smith, bleed to sixteen ounces, and bleed again and again 'to the subdual of inflammation.' The marks of inflammation are as yet, however, indistinct, and inflammation itself not yet developed, according to Dr. Smith's own ideas of the theory of Fever, which perfectly accord with ours. Besides, the only external signs we can ever have of an *internal* inflammation are Pyrexia and pain, and the latter of these is frequently absent in Fever; and the former, Dr. Smith informs us, and we cordially agree with him, can never be cut short by a stroke of art. We fear, therefore, that as a practical canon the subdual of inflammation is one of vague and uncertain application. It is true, indeed, that pain of the head is most generally present, but we apprehend that no means which fail in removing Pyrexia will succeed in banishing, that is completely, head-ache. Again, in this stage of Fever we really possess no positive means of distinguishing, *à priori*, the mildest from the most intense and dangerous types of Fever; but here we are obliged to apply the same canon to all." 64.

Dr. O'Brien conceives that large detractions of blood in the incipient stage of fever accord only with that doctrine which supposes inflammation to be its primary and essential cause. With Dr. Smith's theoretical principles, Dr. B. accords—he only differs from him in the extent to which blood-letting should be carried in the incipient stage of fever—"an extent neither consonant with Dr. Smith's own theory, nor supported by sufficient evidence." The following is Dr. O'Brien's practice in typhoid fever unmixed apparently with topical inflammation.

"In the first stage of Fever, the author's practice is merely palliative; he is satisfied with administering a moderate emetic or purgative, enjoining rigid abstinence and confinement to bed; if possible, a warm bath, and he waits a little until a further development of the disease shall have given a probable insight into its nature and type. As soon as re-action has commenced, if it be vivid, and accompanied by increased heat, flushed countenance, frequent and full pulse, blood-letting is then resorted to.—A single venesection of ten or twelve ounces is at first practised, and if this prove insufficient to reduce the pulse, the heat and flush of the skin, and the general excitement, the process is repeated; but beyond this, unless under very peculiar circumstances, the author seldom thinks it safe to proceed. Occasionally, though certainly rarely, a single bleeding, as above, has brought the Synochoid stage of this Fever to a conclusion, and rapidly converted it into the Typhoid. This transition is well illustrated by the case of Farrel, No. 15, above recorded. When this patient was first presented to the author, his Fever was of a strongly marked Synochoid character, with strong and full pulse, flushed countenance, burning skin, and head-ache. He was bled immediately to the amount of ten ozs., and a weak solution of tart. emetic was administered. On the following day, the author was not a little surprised to find his pulse irregular, small, weak, and frequent; his skin of a dusky hue, and covered with large petechiæ; his countenance muddy and Typhoid; and his intellect, which the day before was perfect, now much impaired.—This patient ultimately recovered, after a protracted and doubtful struggle. Another illustration will shew the uncertain and versatile character of the disease we are considering; in the next bed to Farrel, and admitted on the same day, lay a patient named Ryan, nearly also of the same age. The symptoms and general appearance of the two patients were so strikingly similar, that they made a strong impression on my mind; they were treated of course exactly in the same manner; but what was the event? On the following day Farrel was found in the state above described, and Ryan was convalescent. In the month of December, 1829, a patient named Reilly, a grocer's clerk, young, was admitted into the author's ward; with him a note was sent to the Hospital addressed to the apothecary, from a Medical Gentleman, who attended him previous to admission, stating, that he had been bled to sixteen ounces, and that the blood was buffed and cupped. His case wore the Synochoid character, but his head was not yet materially affected; and he was bled again by my direction to twelve ozs.;—on the following day I found him in a paroxysm of maniacal delirium, the most violent I ever witnessed; yet, with a pulse so small, weak, and irregular, that general bleeding could not again be hazarded. This patient was treated by the repeated application of leeches to the head and by the cold affusion; and after a phrenzy nearly of three days' continuance, perfectly recovered from a Fever, which, though marked by symptoms far more violent, was not half the length of Farrel's Fever, before mentioned. I adduce those Cases to show the infinite variety of forms this disease assumes, and the fallacy of conclusions drawn from particular cases; they will also serve to illustrate the author's practice in the Synochoid stage of Synochus. His mode of applying the cold affusion to the head is as follows:—The patient is supported by one or two nurses, while his head projects beyond the bed over a pail or tub placed near its side, another assistant then pours a stream of cold spring water from a considerable height on the crown of the head, previously shaven and denuded, from a large ewer or jug.—The shock is very powerful, and some caution even will be necessary, to prevent the respiration from being suspended. Dr. Smith recommends another mode of applying the cold affusion, which is still more powerful, but less convenient; namely, the patient being seated in a large tub near the bed-side, a man standing on a table by the side of the tub, pours from an elevation as high as his arm can reach, a full stream of cold water out of the pipe of a watering-pot, deprived of the rose, on his naked head. If this mode of applying the cold affusion be adopted, we should think it would add to its efficacy to immerse the patient's feet in warm water. The next class of medicines available in the Synochoid stage of Fever are purgatives and diaphoretics; the ordinary purgatives are castor oil, or infusion of senna and sulphate of magnesia. The author seldom uses calomel as a purgative, a little influenced, he owns, by the clamour of the French writers against irritating purgatives;—calomel, however, is very generally employed in minute doses, in combination with antimonial powder as a diaphoretic; thus, a grain or half a grain of calomel, with two grains of antimonial powder are given on the days in which purgation is omitted, twice or thrice; whether this combination produces its salutary effects, by acting on the skin, or by improving the secretions of the alimentary canal, the author cannot determine; but he has invariably found it a useful medicine. In cases, however, of abdominal irritation, it seems inapplicable, and ought to be withheld." 68.

We need not dwell on the stage of exhaustion in typhoid fever. Dr. O'Brien's practice is one rather of vigilant observation than of active treatment. Generally speaking, however, "it is tonic and moderately stimulant." Gentle laxatives, blisters, &c. are employed; but wine and opium have not answered his expectations, and on them he places little or no reliance.

We must now close our analysis of Dr. O'Brien's report. It is valuable, as proceeding from an institution where experience, on a large scale, forms the basis and affords the data.

